# Expression of GATA-3 During Lymphocyte Differentiation and Mouse Embryogenesis

**DOI:** 10.1155/1992/27903

**Published:** 1992

**Authors:** Mariëtte Oosterwegel, Janneke Timmerman, Jeffrey Leiden, Hans Clevers

**Affiliations:** 1 Department of Immunology University Hospital Utrecht The Netherlands; 2 Departments of Medicine and Pathology University of Chicago Chicago Illinois USA

**Keywords:** GATA-3, transcription factors, T-cell differentiation, lymphoid differentiation, embryogenesis

## Abstract

The GATA family of C4 zinc-finger transcription factors has been implicated in tissuespecific gene regulation in birds and mammals. One of the members of this family, GATA-3, is reportedly expressed specifically in the T-cell lineage, where it interacts with GATA motifs in the TCR-*αα*, TCR-β/, and TCR-δ enhancers, thereby controlling the T-cell phenotype. To evaluate the differentiation control properties of GATA-3, we have now documented its expression pattern during lymphoid differentiation and murine embryogenesis.

The onset of GATA-3 expression in the lymphoid lineage was studied in a panel of lymphoid (precursor) cell lines by Northern blot analysis. GATA-3 was uniquely expressed in T-lineage lymphocytes expressing TCR and CD3 genes; it was absent from TCR/CD3 mRNA-negative prothymocytes and from all B-lineage cells.

In order to obtain information on the expression of GATA-3 outside the immune system, *in situ* hybridization was performed on mouse embryos on day 11.5-14.5 of gestation. GATA-3 mRNA was detected in fetal thymus and in erythroid cells. Outside the haemopoietic system, we detected GATA-3 mRNA throughout the central nervous system, in kidney, in the epidermis, lens fibers, the inner ear, whisker follicles, and in the primary palate.

These data provide new clues about the potential role of GATA-3 during mouse development, and will aid the interpretation of currently ongoing gene knockout experiments.


*Developmental Immunology*, 1992, Vol. 3, pp. 1–11
Reprints available directly from the publisher
Photocopying permitted by license only

© 1992 Harwood Academic Publishers GmbH
Printed in the United States of America

### Expression of GATA-3 During Lymphocyte Differentiation and Mouse Embryogenesis

MARIËTTE OOSTERWEGEL,† JANNEKE TIMMERMAN,† JEFFREY LEIDEN,‡ and HANS CLEVERS*†

†*Department of Immunology, University Hospital, Utrecht, The Netherlands*
‡*Departments of Medicine and Pathology, University of Chicago, Chicago, Illinois*

The GATA family of C4 zinc-finger transcription factors has been implicated in tissue-specific gene regulation in birds and mammals. One of the members of this family, GATA-3, is reportedly expressed specifically in the T-cell lineage, where it interacts with GATA motifs in the TCR-$\alpha$, TCR-$\beta$, and TCR-$\delta$ enhancers, thereby controlling the T-cell phenotype. To evaluate the differentiation control properties of GATA-3, we have now documented its expression pattern during lymphoid differentiation and murine embryogenesis.

The onset of GATA-3 expression in the lymphoid lineage was studied in a panel of lymphoid (precursor) cell lines by Northern blot analysis. GATA-3 was uniquely expressed in T-lineage lymphocytes expressing TCR and CD3 genes; it was absent from TCR/CD3 mRNA-negative prothymocytes and from all B-lineage cells.

In order to obtain information on the expression of GATA-3 outside the immune system, *in situ* hybridization was performed on mouse embryos on day 11.5–14.5 of gestation. GATA-3 mRNA was detected in fetal thymus and in erythroid cells. Outside the haemopoietic system, we detected GATA-3 mRNA throughout the central nervous system, in kidney, in the epidermis, lens fibers, the inner ear, whisker follicles, and in the primary palate.

These data provide new clues about the potential role of GATA-3 during mouse development, and will aid the interpretation of currently ongoing gene knockout experiments.

KEYWORDS: GATA-3, transcription factors, T-cell differentiation, lymphoid differentiation, embryogenesis

### INTRODUCTION

Mature T and B lymphocytes are thought to arise from pluripotent stem cells through a common lymphoid progenitor and several discrete precursor B- and T-cell stages. Each step in this differentiation pathway is characterized by specific changes in cellular phenotype, which in turn is determined by the complement of actively transcribed structural genes. Control of the expression of such sets of genes is exerted by developmentally active transcription factors. The regulated activity of these transcription factors is believed to be central to the control of phenotype during differentiation (Maniatis et al., 1987; Atchinson, 1988; Johnson and McKnight, 1989).

One family of transcription factors implicated in tissue-specific gene regulation is the GATA family. All GATA proteins recognize the GATA consensus sequence and contain a C4 zinc-finger motif. GATA-3, one of the members of this family, is a candidate control gene implied to be involved in T-cell differentiation (Ho et al., 1991; Ko et al., 1991; Marine and Winoto, 1991). The prototypic factor of this family, GATA-1 (previously termed GF-1, EryF1, or NF-E1), was originally cloned from murine erythroid cells, based on its affinity for the GATA motif in the $\beta$ globin gene (Tsai et al., 1989). Its expression was then analyzed in detail and was observed to be restricted to cells of the erythroid lineage, mast cells and megakaryocytes (Martin et al., 1990). Subsequently, three homologous genes (NF-E1a, NF-E1b, and NF-E1c, renamed GATA-1, GATA-2, and GATA-3) were cloned from chicken

*Corresponding author.

erythrocytes under low-stringency conditions, using the murine GATA-1 cDNA (Yamamoto et al., 1990). Based on homology, cDNA clones encoding human GATA-1, -2, and -3 and murine GATA-3 have since been isolated (Zon et al., 1990; Ho et al., 1991; Ko et al., 1991; Marine and Winoto, 1991; Dorfman et al., 1992). All three proteins have been demonstrated to transactivate transcription from a minimal TATA-box promoter adjacent to a multimerized GATA motif (Ho et al., 1991; Marine and Winoto, 1991; Dorfman et al., 1992).

The expression pattern of the three chicken GATA genes has been studied by Northern analysis. GATA-3, the subject of the present study, is thus reportedly expressed in chicken erythroid cells, T-lineage cells, fetal brain and adult kidney (Yamamoto et al., 1990). Similar studies performed in human and mouse so far indicate that GATA-3 expression is restricted to T-lineage lymphocytes (Ho et al., 1991; Ko et al., 1991; Marine and Winoto, 1991). In line with this observation, functional GATA motifs have been detected in the T-cell-specific enhancers of the TCR-$\alpha$, -$\beta$, and $\delta$ genes (Ho et al., 1991; Ko et al., 1991; Marine and Winoto, 1991).

In order to evaluate the possible differentiational control properties of GATA-3, we have now analyzed in detail the onset of GATA-3 expression during murine lymphocyte differentiation. To that end, Northern analysis was performed on a panel of well-defined lymphoid (precursor) cell lines. We arbitrarily distinguish three different phenotypical stages in early T-cell development with regard to rearrangement and expression of the genes encoding the constituent chains of the TCR/CD3 complex. Prothymocytes express Thy1 and CD2. They do not express TCR or CD3 genes other than CD3-$\gamma$ and have undergone no TCR gene rearrangements. Prethymocytes express all CD3 chains as well as partially rearranged TCR chains. Thymocytes/T cells have fully rearranged TCR genes, and carry TCR/CD3 complexes on the cell surface. CD4 and CD8 first appear on TCR/CD3-surface-positive thymocytes. Corresponding stages have been defined in the B-cell lineage (Alt et al., 1981).

As chicken GATA-3 was found expressed outside the immune system, and as we have observed by Northern analysis that high expression of GATA-3 occurs in the murine kidney (J. Leiden, unpublished), we decided to extend our study by *in situ* hybridization analyses during murine embryogenesis.

### RESULTS

In order to determine the onset of GATA-3 expression in the T-cell lineage, Northern analysis was performed on a panel of precursor and mature lymphoid cell lines. To that end, RNA was isolated from a panel of murine prothymocytes, prethymocytes, and thymocytes/T cells, pro-B, pre-B, and mature B cells and control fibroblast cell lines. The prothymocyte and prethymocyte cell lines will be described in detail elsewhere (A. Kruisbeek, in preparation). Briefly, prothymocytes express Thyl, CD2, and CD3-$\gamma$, whereas no CD3-$\delta$, CD3-$\varepsilon$, TCR$\alpha$, TCR-$\beta$, CD4, or CD8 mRNA is present yet. All other T-cell lines transcribe the genes encoding the constituent chains of the TCR/CD3-complex (see also Materials and Methods).

As shown in Fig. 1D, GATA-3 was abundantly expressed in prethymocytes and thymocytes/T cells. No GATA-3 was detectable in prothymocytes, B-lineage cells, or fibroblasts. CD3-$\varepsilon$ and TCR-$\alpha$ expression coincided with that of GATA-3 (Figs. 1B and 1C); CD3-$\gamma$ was already expressed by the prothymocyte cell lines, indicative of their T-lineage origin. Remarkably, CD3-$\gamma$ mRNA was also found in the myeloma cell lines Ag8 and Ns1 (Fig. 1A).

We next studied expression of GATA-3 by *in situ* hybridization of murine embryos. Sections of day 11.5, 12.5, and 14.5 embryos were hybridized with a 3' untranslated antisense RNA probe under high stringency conditions. Figure 2 depicts a typical experiment using the GATA-3 probe on a midsagittal section of a day 14.5 embryo. Most sites of expression are visible on the whole-embryo section. In what follows, we discuss the expression of GATA-3 per individual organ system, illustrated by higher-magnification pictures where appropriate.

### Thymus

On day 12.5, when the thymus was first visible, GATA-3 mRNA could be detected in this organ (Fig. 3A). A moderate level of GATA-3 mRNA was also apaprent at day 14.5 (Fig. 2). Most likely, thymocytes were responsible for the

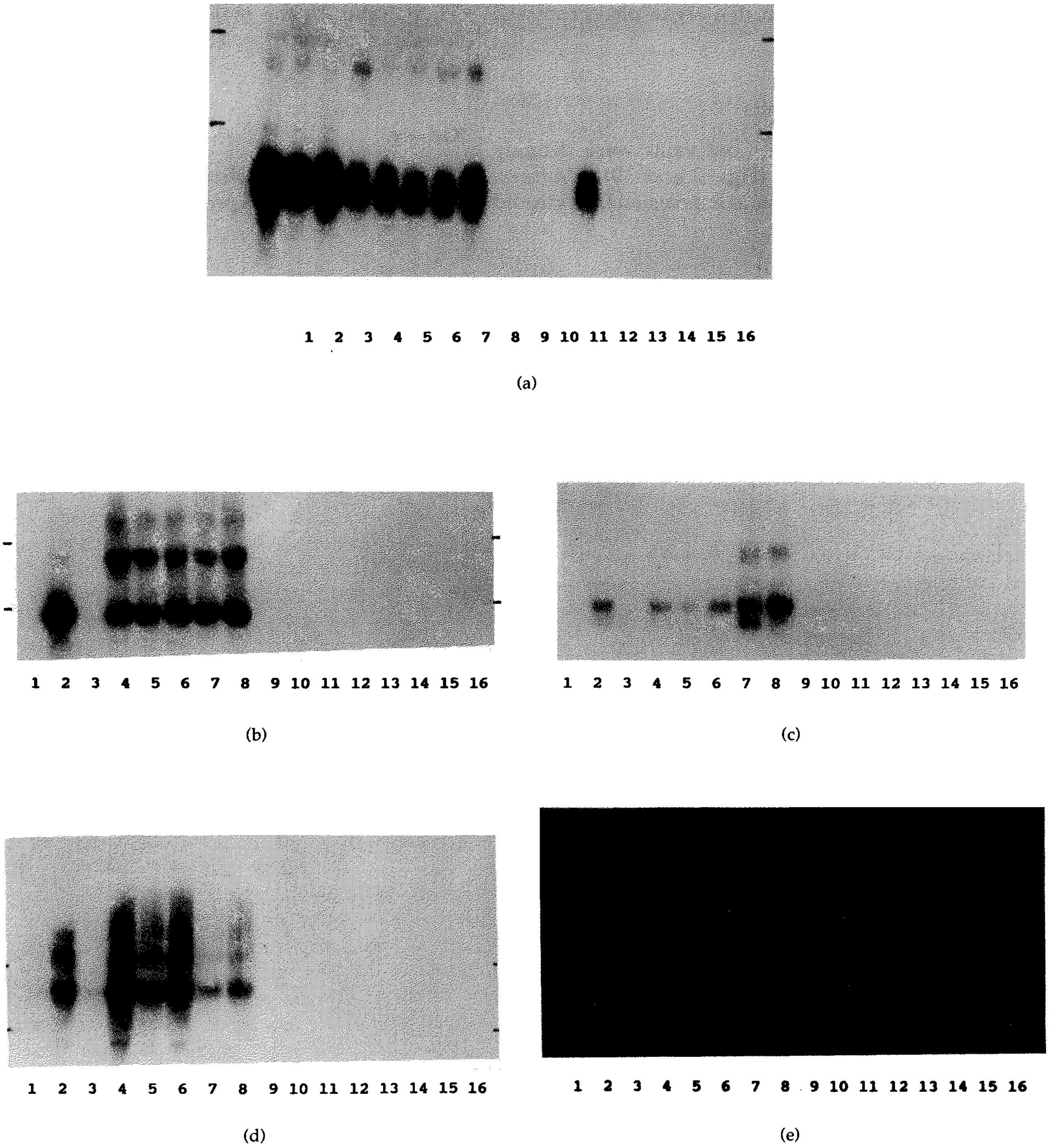

FIGURE 1. Northern blot analysis of CD3-$\gamma$, CD3-$\varepsilon$, TCR-$\alpha$, and GATA-3 in a panel of lymphoid (progenitor) cells. Northern blots were probed for (A) CD3-$\gamma$, (B) CD3-$\varepsilon$, (C) TCR-$\alpha$, and (D) GATA-3. Ticks mark the ribosomal bands. Equal amounts of RNA were analyzed, as is evident from (E) the ethidium bromide-stained nitrocellulose blot: (1) 34.1.E and (3) 35.1.E are day 14 fetal thymus-derived prothymocyte cell lines; (2) 34.1.L and (4) 35.1.L are day 14 fetal thymus-derived prethymocyte cell lines; (5) A1 and (6) B2 are day 17 fetal thymus-derived T cell lines; (7) EL-4 and (8) BS5147 are mature TCR-$\alpha\beta$ T-cell lines; (9) 38B9 is a pro-B-cell line; (10) 40E1 and (11) 1881 are pre-B-cell lines; (12) Ag8 and (13) Ns1 are myeloma-derived B-cell lines; (14) NIH-3T3, (15) L cells and (16) 3T6 are fibroblast cell lines.

observed GATA-3 signal. However, it could not be excluded that thymic epithelium or other structural elements in this organ also expressed GATA-3.

### Erythroid Lineage Cells

The lumina of arteries and veins were strongly positive for GATA-3 (Figs. 2 and 3B). We interpreted this observation as expression in erythroid cells, abundantly present in these blood vessels. Consistent with this observation, the fetal liver (Figs. 2 and 4), the major site of fetal erythropoiesis, and the heart (Fig. 3A) also expressed GATA-3.

### Kidney

At all days tested, prominent expression was observed in kidney. Expression was first detected

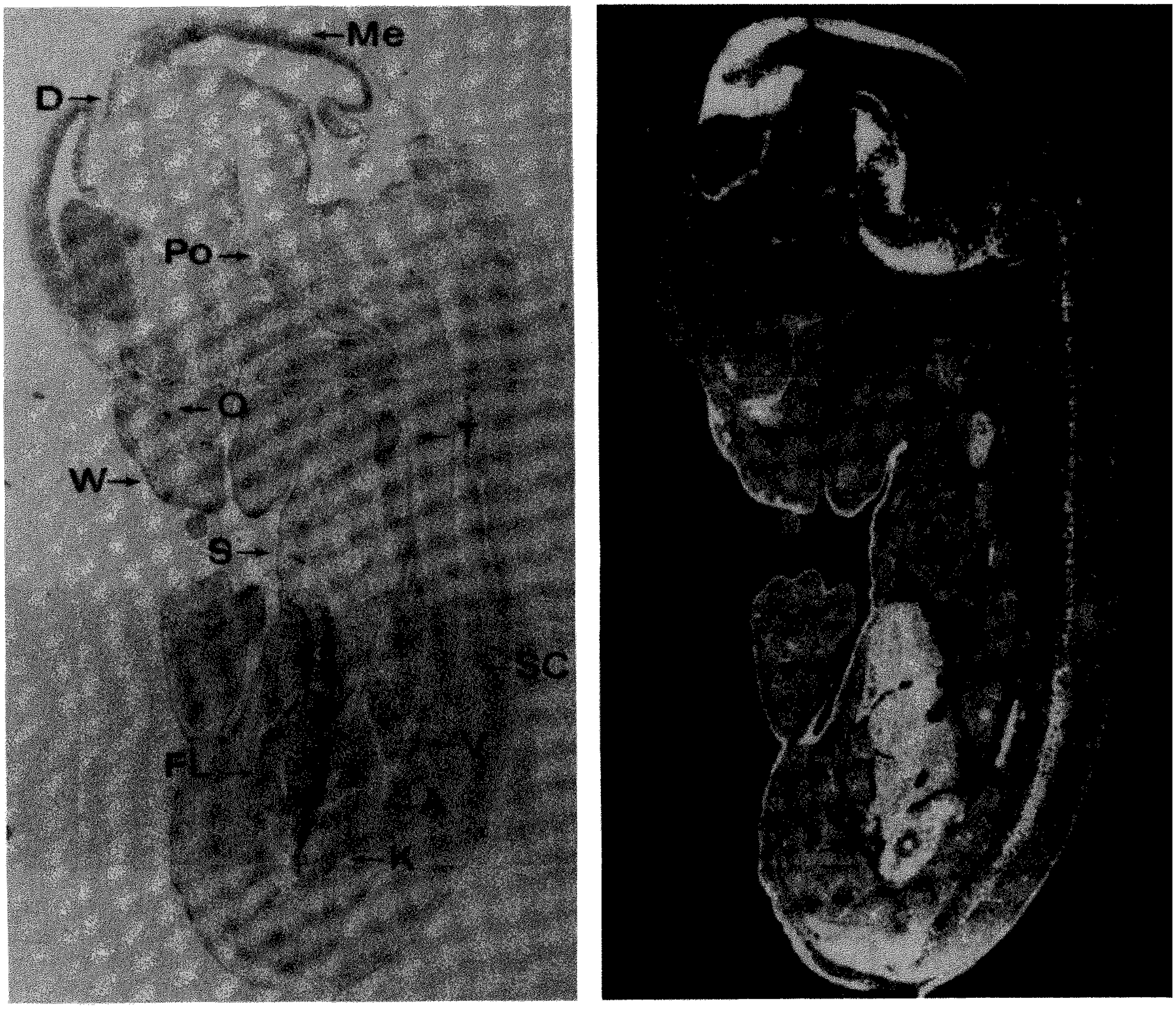

FIGURE 2. *In situ* hybridization for GATA-3 on midsagittal sections of day 14.5 murine embryos. Data presented here represent examples of multiple *in situ* experiments on serial sections of independent embryos. Bright field (left) and the accompanying dark field (right) pictures are shown. GATA-3 was observed in the collecting tubules of the kdiney (=K) and in the medulla of the adrenal gland (=A); in the thymus (=T) and the fetal liver (=FL); in dorsal blood vessels (=V); in the central nervous system, in the diencephalon (=D), mesencephalon (=Me), the metencephalon-derived pons (=Po), and in the spinal cord (=SC); in the epidermis (=S), whisker follicles (=W), and in the mesenchymal cells that form the periorbital region (=O).

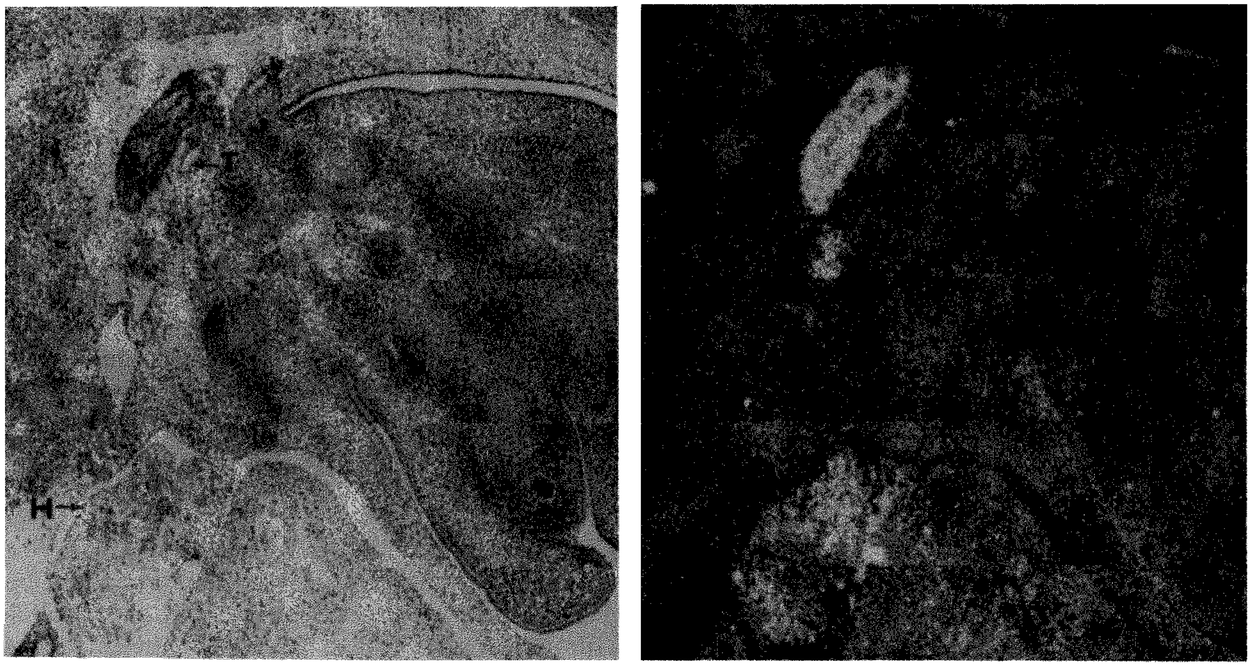

(a)

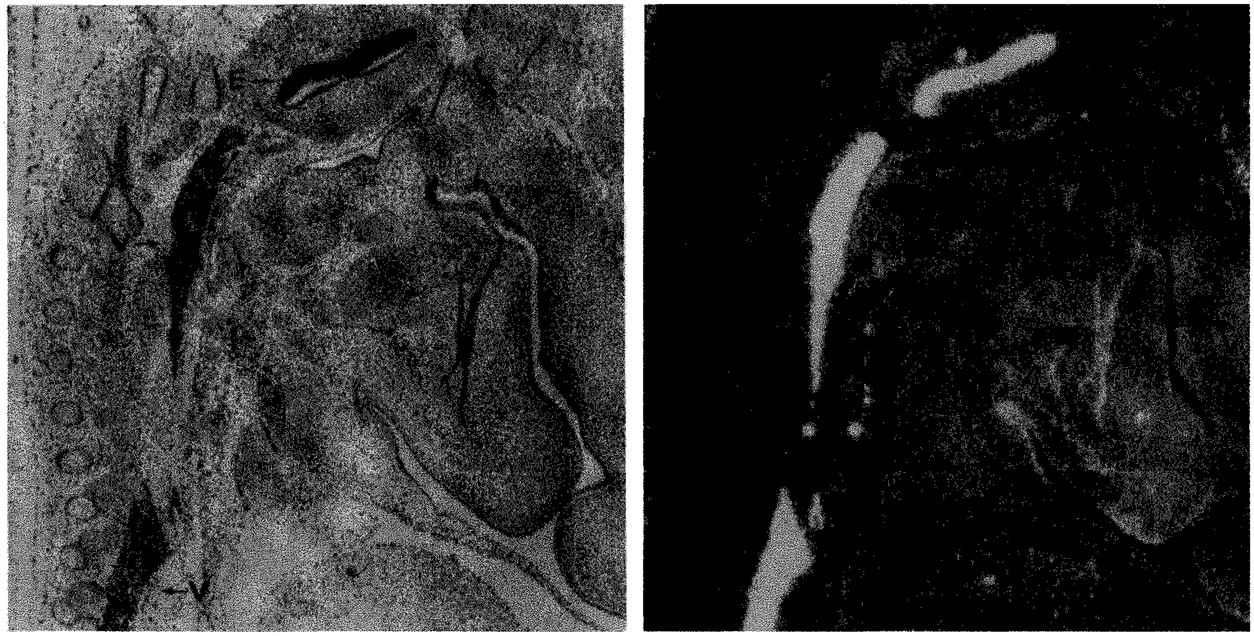

(b)

FIGURE 3. GATA-3 expression in thymus and in erythroid cells. In the haemopoietic system, GATA-3 was expressed in T cells and in cells of the erythroid lineage. (A) At day 12.5, the thymus (=T) and erythroid cells in the heart (=H) contained GATA-3 mRNA. (B) GATA-3 was detected in erythroid cells in blood vessels (=V). In addition, cochlear epithelial cells of the inner ear (= E) expressed GATA-3.

in the mesonephric duct (day 11.5, not shown) and was strictly localized in the collecting tubuli at later stages (Fig. 4). These observations confirmed Northern blot experiments performed on adult kidney (J. Leiden, unpublished).

### Adrenal Gland

GATA-3 mRNA was detected at significant levels in the neural crest-derived medulla of the adrenal gland. No GATA-3 was detectable in the cortex of this endocrine organ, which is of mesodermal origin (Fig. 4).

### Central Nervous System

High-level expression of GATA-3 was observed throughout the brain and the spinal cord at all days examined. At day 11.5, exclusive expression was detected at the ventro-lateral site of the neural tube (not shown). At day 14.5, large areas of the diencephalon, the mesencephalon, and the pons in the metencephalon were positive, as was the ventral site of the spinal cord (Fig. 2).

### Epidermis and Skin-Derived Sensory Organs

Expression of GATA-3 in the epidermis was observed at all days, but increased during gestation (Figs. 2 and 5). At day 11.5, lens fibers contained GATA-3 mRNA (Fig. 5A). This signal became undetectable at later stages. At day 12.5, mesenchymal cells of the periorbital region started expressing GATA-3 and still did at day 14.5 (Figs. 2, 5B, and 5C). At day 12.5 and 14.5, whisker follicles (Figs. 5B and 5C), representing invaginated skin, and cochlear epithelial cells of the inner ear (Fig. 3B) expressed high levels of GATA-3 mRNA.

### Palate

On day 12.5, the primary palate expressed high levels of GATA-3. At this stage, endochondral ossification initiates and the palate closes (Fig. 6).

### DISCUSSION

The present report describes the expression of GATA-3 during lymphocyte differentiation and murine embryogenesis. In a panel of lymphoid (precursor) cell lines, GATA-3 expression was found restricted to the T-cell lineage and corresponded with the onset of expression of the target genes TCR-$\alpha$ and TCR-$\beta$. These observations are consistent with the proposed regulatory role of GATA-3 in T-cell differentiation. An additional argument for such a function is the conservation between chicken, mouse, and man of GATA-3 sequence and expression in T cells (Ko et al., 1991). Gene knockout experiments

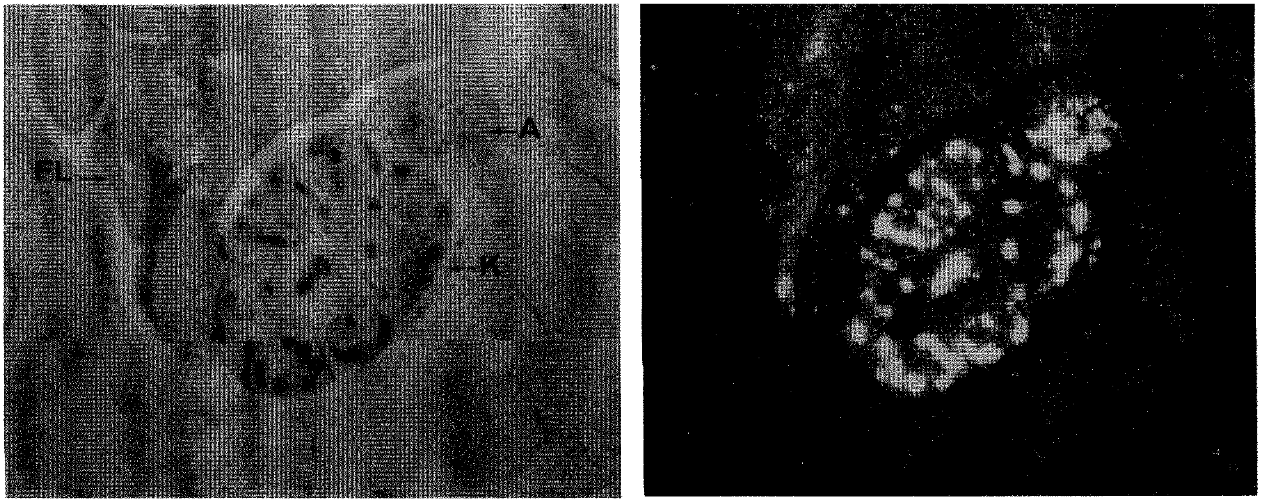

FIGURE 4. Expression of GATA-3 in kidney and adrenal gland. At day 14.5, high-level expression of GATA-3 was observed in the collecting tubules of the kidney (=K) and in the medulla of the adrenal gland (=A). Fetal liver (=FL) was also positive, most likely due to the abundant presence of erythroid precursor cells.

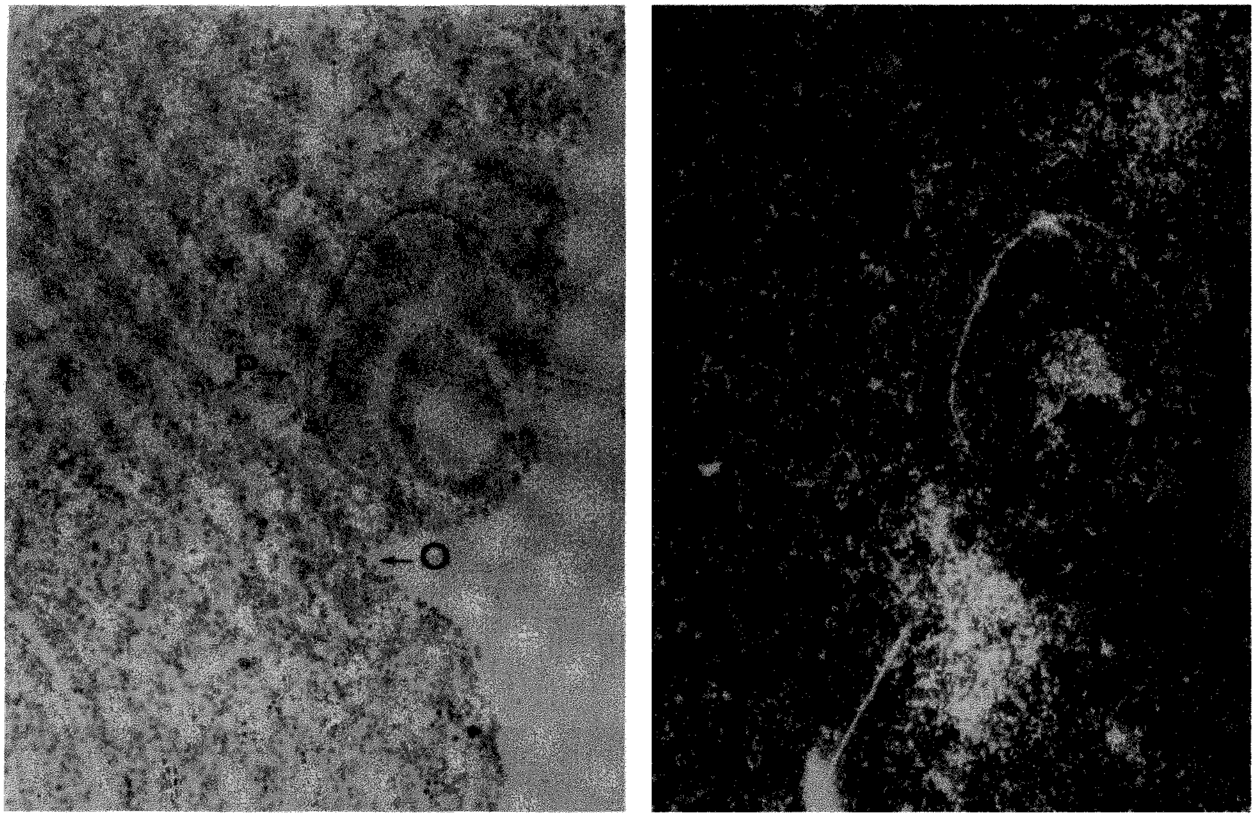

(a)

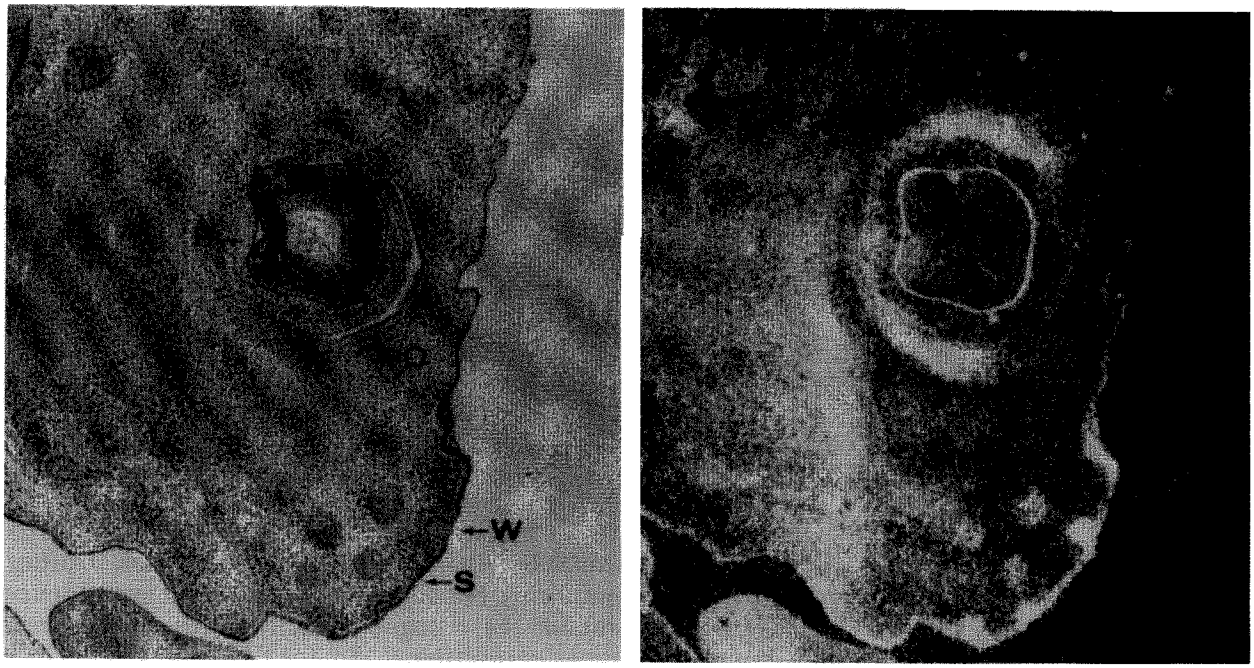

(b)

FIGURE 5.

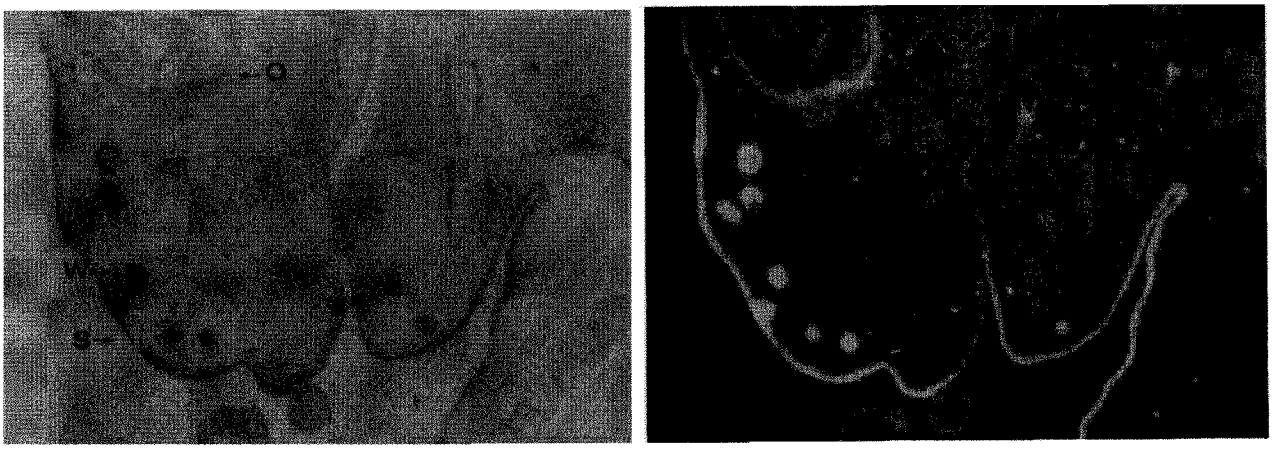

(c)

FIGURE 5. Expression of GATA-3 in epidermis and sensory organs derived from invaginated skin. At day 11.5, GATA-3 mRNA was present in lens fibers (=L). Mesenchymal cells that form the perioribital region (=O) expressed GATA-3 at all days. Pigment cells in the retina (=P) provide a nonspecific signal in dark field microscopy. From day 12.5, epidermis (=S) and whisker follicles (=W) expressed GATA-3. (A) Day 11.5, (B) day 12.5, and (C) day 14.5

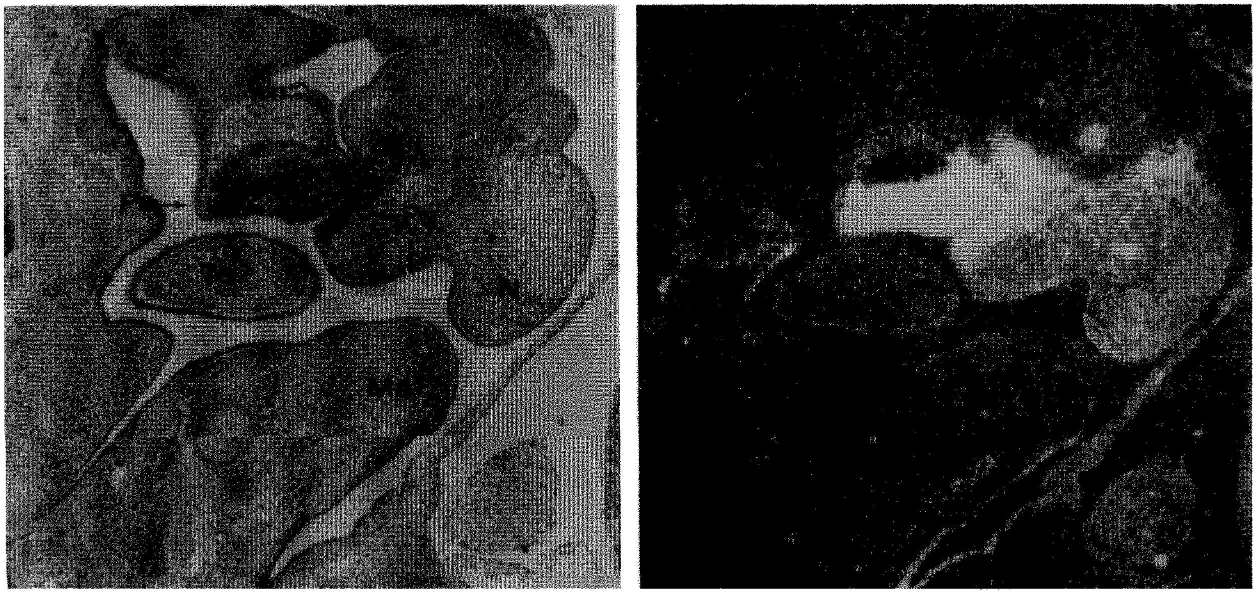

FIGURE 6. GATA-3 expression in palate. At day 12.5 of gestation, GATA-3 mRNA was detected in the primary palate (=Pa). N= nasal fold, To=tongue, and Ma=mandible.

have demonstrated that the closely related GATA-1 gene is essential for normal erythroid differentiation (Pevny et al., 1981). It is tempting to speculate that GATA-3 is equally important for the T-cell lineage.

*In situ* hybridization of sections of murine embryos at days 11.5, 12.5, and 14.5 revealed multiple sites of GATA-3 expression during ontogeny. No common denominator was apparent for the ontogenic origin of the various cell types transcribing the GATA-3 gene. GATA-3 mRNA was thus observed in cell types of (neuro-)ectodermal (central nervous system, epidermis, and skin-derived organs, medulla of the adrenal gland) and mesodermal origin (thymocytes, erythroid cells, kidney, and palate). The expression of murine GATA-3 in erythroid cells, fetal brain and kidney as described here, is consistent with the expression pattern reported for chicken GATA-3 (Yamamoto et al., 1990).

At first glance, the complex expression pattern of GATA-3 appears to be at variance with an important role for this gene in T-cell differentiation. However, it is a common finding that putative tissue-specific transcription factors display a wider tissue distribution than the structural target gene originally used to identify the factor. For example, the POU-domain genes Oct-2 and Pit-1, which are believed to control specific expression of genes in the B-cell lineage and in pituitary cells, respectively, both are expressed in the developing nervous system (Xi et al., 1989). In addition, LF-B1 is thought to be involved in liver-specific gene expression, but is expressed in a wide variety of other tissues (Baumhueter et al., 1990). It is generally assumed that the exquisite expression of target genes is the result of the combinatorial activity of a number of positively and negatively acting transcription factors with restricted tissue distributions (Johnson and McKnight, 1989). Thus, a limited number of transcription factors may be utilized in varying combinations to produce a large variety of target gene expression patterns. From this, it appears likely that GATA-3 acts in concert with additional transcription factors to control differentiation within the T-cell lineage.

Other transcription factors potentially involved in T-cell differentiation are TCF-1, LEF-1, and Ets-1 (Leiden, 1992). Amongst these factors, the expression of TCF-1 in the lymphoid lineage (Oosterwegel, unpublished) most closely correlates with that of GATA-3. TCF-1 contains an HMG box DNA-binding domain and recognizes sequence motifs in the CD3-$\varepsilon$, TCR-$\alpha$, TCR-$\beta$, and TCR-$\delta$ enhancers (Oosterwegel et al., 1991; van de Wetering et al., 1991). The expression of TCF-1 during mouse embryogenesis is equally complex as that of GATA-3. However, the only cell type that coexpresses TCF-1 and GATA-3 is the thymocyte. Therefore, we hypothesize that a specific combination of TCF-1 and GATA-3 is important for proper differentiation of T-lineage cells. Knockout experiments in the mouse germ line will shed light on the differentiation control properties of GATA-3. The expression data reported here will serve as a starting point for the evaluation of such experiments.

### MATERIALS AND METHODS

### Cell Lines

Cells were grown in RPMI-1640 supplemented with 5% fetal calf serum and antibiotics. All cell lines were of murine origin. Pro- and prethymocyte cell lines were derived from day 14 fetal thymus organ culture, and transformed with a retroviral *myc-raf* oncogene construct (J2) (A. Kruisbeek, in preparation). Prothymocytes, 34.1.E and 35.1.E, express CD2, Thyl, and the CD3-$\gamma$ gene. They are CD4/CD8-negative and their T-cell receptor genes are in germ-line configuration. No CD3-$\delta$ or CD3-$\varepsilon$ mRNA is detectable. This phenotype is consistent with that described by Palacios and Pelkonen (1988). Prethymocytes, 34.1.L and 35.1.L, express CD3-$\gamma$, CD3-$\delta$, and CD3-$\varepsilon$ mRNA as well as partially rearranged TCR-$\beta$ mRNA and TCR-$\alpha$ mRNA; they are surface TCR/CD3 and CD4/CD8-negative. T-cell lines derived by transformation of day 17 fetal thymocytes, A1 and B2, display a mature T-cell phenotype, including the surface expression of the TCR/CD3 complex and CD4 and CD8. 1881 is a $\mu$-negative mutant pre-B cell line, 38B9 represents a pro-B cell line; 40E1 pre-B cells express $\mu$ and $\lambda5$. 38B9 and 40E1 are derived from fetal liver, and 1881 is derived from adult bone marrow by transformation with Abelson virus (Alt et al., 1981). All other cells were obtained from the American Type Culture Collection. Briefly, EL-4 is a mature TCR-$\alpha/\beta$ surface-positive T-cell line. The phenotype of BW5147 is similar to that of EL-4, but as a result of the absence of CD3-$\delta$ mRNA, no surface expression of the TCR/CD3 complex occurs. Ag8 and Ns1 are B-lineage cell lines (non-Ig secreting myeloma cells). NIH-3T3, 3T6, and L cells are fibroblast cell lines.

### Northern Blot Analysis

Northern blot analysis was performed according to standard procedures. RNA was isolated by lithium chloride/urea precipitation. For each sample, 10 $\mu$g of RNA was run on a 1.2% agarose gel containing 6% formaldehyde. RNA was blotted onto reinforced nitrocellulsoe filters (BAS 85, 0.45 $\mu$m, Schleicher and Schuell). Hybridization mix contained 5×SSPE, 5×Denhardt, 0.1% SDS, 50% formamide, 10% dextran sulfate, and 100 $\mu$ g/ml salm sperm DNA. Hybridization was performed overnight at 42°C. High stringency washing was performed using 0.2×SSC/0.1% SDS at 65°C. Kodak X-AR5 films were exposed up to 7 days with amplifying screens at –80°C.

### cDNA Probes

Human GATA-3 was cloned from an HPB-ALL T-cell cDNA library by oligo probing based on published sequence (Leiden, 1992). Subsequently, murine cDNA clones were obtained from an EL-4 phage cDNA library under conditions of low stringency using the human cDNA clone as a probe. The identity of all cDNAs was confirmed by sequencing. For Northern blotting, DNA probes were labeled by random oligonucleotide priming (Pharmacia). For CD3-$\gamma$, CD3-$\varepsilon$, and TCR-$\alpha$, full-length cDNA inserts were used. For GATA-3, an 800-bp fragment, extending downstream from the HindIII site at bp 1234 (Ko et al., 1991), was used.

### *In situ* Hybridization

*In situ* hybridization was performed essentially as described by Wilkinson et al. (1987) with modifications introduced by Kress et al. (1990). Briefly, dissected embryos were fixed 4 to 18 hr in 4% paraformaldehyde, dehydrated in ethanol and butanol, and embedded in paraffin. Embryos were sectioned at 6 $\mu$m thickness and mounted on 3-aminopropyl-triethoxy-silane-coated slides. Slides were deparaffined, digested with proteinase K, acetylated with triethanolamine, dehydrated and hybridized overnight at 55°C. Subsequently, high stringency washing was performed in 50% formamide/2×SSC/10 mM DTT at 65°C. Slides were treated with ribonuclease A, rinsed in 2×SSC, 0.1×SDS, dehydrated and dipped in Ilford emulsion G.5. After drying, the slides were exposed for up to 10 days at 4°C, developed in Kodak D-19, and fixed with Kodak LX-24.

### RNA Probe

An antisense radiolabeled RNA probe was generated from GATA-3 cDNA subcloned in pBluescript SK, using T7 RNA polymerase and $^{35}$S-UTP (Stratagene). The construct was linearized with BglII to yield a 458-bp-labeled probe, covering 3' untranslated sequence. The probe was used for hybridization at 1× $10^5$ c.p.m./$\mu$l.

### ACKNOWLEDGMENTS

We thank Dr. J. Deschamps and Dr. O. Destree, R. Vogels, P. Lanse, M. Wijgerde and several other people from the Hubrecht Laboratory for technical assistance and helpful discussions; Dr. A. M. Kruisbeek and Dr. J. F. Kearney and A. Hamilton for kindly providing the thymocyte cell lines, the pre-B and BW5147 cell lines, respectively; Drs. F. Meijlink, S. Verbeek, and J. van de Winkel for critically reading the manuscript; T. van Rijn, R. Scriwanek, and F. Vervoordeeldonk for photography.

*(Accepted July 19, 1992)*

### REFERENCES

Alt F., Rosenberg N., Lewis S., Thomas E., and Baltimore D. (1981). Organization and reorganization of immunoglobulin genes in A-MuLV-transformed cells: Rearrangement of heavy but not light chain genes. Cell **27:** 381–390.

Atchinson M.L. (1988). Enhancers: Mechanism of action and cell specificity. Annu. Rev. Cell Biol. **4:** 127–154.

Baumhueter S., Mendel D.B., Conley P.B., Kuo C.J., Turk C., Graves M.K., Edwards C.A., Courtois G., and Crabtree G.R. (1990). HNF-1 shares three sequence motifs with the POU-domain proteins and is identical to LF-B1 and APF. Genes and Develop. **4:** 372–379.

Dorfman D.M., Wilson D.B., Bruns G.A.P., and Orkin S.H. (1992). Human transcription factor GATA-2. J. Biol. Chem. **267:** 1279–1285.

Ho I., Voorhees P., Marin N., Oakley B.K., Tsai S., Orkin S.H., and Leiden J.M. (1991). Human GATA-3: A lineage restricted transcription factor that regulates the expression of the T cell receptor $\alpha$ gene. EMBO J. **10:** 1187–1192.

Johnson P.F., and McKnight S.L. (1989). Eukaryotic transcriptional regulatory proteins. Annu. Rev. Biochem. **58:** 799–839.

Ko, L.J., Yamamoto M., Leonard M.W., George K.M., Ting P., and Engel J.D. (1991). Murine and human T-lymphocyte GATA-3 factors mediate transcription through a cis-regulatory element within the human T-cell receptor $\delta$ gene enhancer. Mol. Cell. Biol. **5:** 2778–2784.

Kress C., Vogels R., de Graaf W., Bonnerot C., Meijlink F., Nicolas J.-F., and Deschamps J. (1990). Hox-2.3 upstream sequences mediate lacZ expression in intermediate mesoderm derivatives of transgenic mice. Development **109:** 775–786.

Leiden J.M. (1992). Transcriptional regulation in lymphocytes. Immunol. Today **13:** 22–30.

Maniatis T., Goodburn S., and Fisher J.A. (1987). Regulation of inducible and tissue-specific gene expression. Science **236**: 1237–1245.

Marine J., and Winoto A. (1991). The human enhancer-binding protein GATA3 binds to several T-cell receptor regulatory elements. Proc. Natl. Acad. Sci. USA **88**: 7284–7288.

Martin D.I.K., Zon L.I., Mutter G., and Orkin S.H. (1990). Expression of an erythroid transcription factor in megakaryocytic and mast cell lineages. Nature **344**: 444–447.

Oosterwegel M., van de Wetering M., Dooijes D., Klomp L., Winoto A., Georgopoulos K., Meijlink F., and Clevers H. (1991). Cloning of murine TCF-1, a T cell-specific transcription factor interacting with functional motifs in the CD3-$\varepsilon$ and the T cell receptor $\alpha$ enhancers. J. Exp. Med. **173**: 1133–1142.

Palacios R., and Pelkonen J. (1988). Prethymic and intrathymic mouse T-cell progenitors. Growth requirements and analysis of the expression of genes encoding TCR/CD3 components and other T-cell-specific molecules. Immunol. Rev. **104**: 5–23.

Pevny L., Simon M.C., Robertson E., Klein W.H., Tsai S., Agati V.D., Orkin S.H. and Constantini F. (1991). Erythroid differentiation in chimaeric mice blocked by a targeted mutation in the gene for transcription factor GATA-1. Nature **349**: 257–260.

Tsai S., Martin D.I.K., Zon L.I., Andrea A.D., Wong G.G., and Orkin S.H. (1989). Cloning of cDNA for the major DNA-binding protein of the erythroid lineage through expression in mammalian cells. Nature **339**: 446–451.

van de Wetering M., Oosterwegel M., Dooijes D., and Clevers H. (1991). Identification and cloning of TCF-1, a T cell-specific transcription factor containing a sequence-specific HMG box. EMBO J. **10**: 123–132.

Wilkinson D.G., Bailes J.A., Champion J.E., and McMahon A.P. (1987). A molecular analysis of mouse development from 8 to 10 days post coitum detects changes only in embryonic globin expression. Development **99**: 493–500.

Xi H., Treacy M.N., Simmons D.M., Ingraham H.A., Swanson L.W., and Rosenfeld M.G. (1989). Expression of a large family of POU-domain regulatory genes in mammalian brain development. Nature **340**: 35–42.

Yamamoto M., Ko L.J., Leonard W., Beug H., Orkin S.H., and Engel J.D. (1990). Activity and tissue-specific expression of the transcription factor NF-E1 multigene family. Genes and Develop. **4**: 1650–1662.

Zon L.I., Tsai S., Burgess S., Matsudaira P., Bruns G.A.P., and Orkin S.H. (1990). The major human erythroid DNA-binding protein (GF-1): Primary sequence and localization of the gene to the X chromosome. Proc. Natl. Acad. Sci. USA **87**: 668–672.

